# Hippo Signaling-Mediated Mechanotransduction in Cell Movement and Cancer Metastasis

**DOI:** 10.3389/fmolb.2019.00157

**Published:** 2020-01-31

**Authors:** Yu-Chiuan Chang, Jhen-Wei Wu, Chueh-Wen Wang, Anna C.-C. Jang

**Affiliations:** ^1^Department of Biotechnology and Bioindustry Sciences, National Cheng Kung University, Tainan, Taiwan; ^2^Institute of Biomedical Sciences, National Sun Yat-sen University, Kaohsiung, Taiwan

**Keywords:** cell migration, *Drosophila*, mechanical force, metastasis, cell size control

## Abstract

The evolutionarily conserved Hippo kinase signaling cascade governs cell proliferation, tissue differentiation and organ size, and can promote tumor growth and cancer metastasis when dysregulated. Unlike conventional signaling pathways driven by ligand-receptor binding to initiate downstream cascades, core Hippo kinases are activated not only by biochemical cues but also by mechanical ones generated from altered cell shape, cell polarity, cell-cell junctions or cell-extracellular matrix adhesion. In this review, we focus on recent advances showing how mechanical force acts through the actin cytoskeleton to regulate the Hippo pathway during cell movement and cancer invasion. We also discuss how this force affects YAP-dependent tissue growth and cell proliferation, and how disruption of that homeostatic relationship contributes to cancer metastasis.

## Introduction

**Figure 1:**
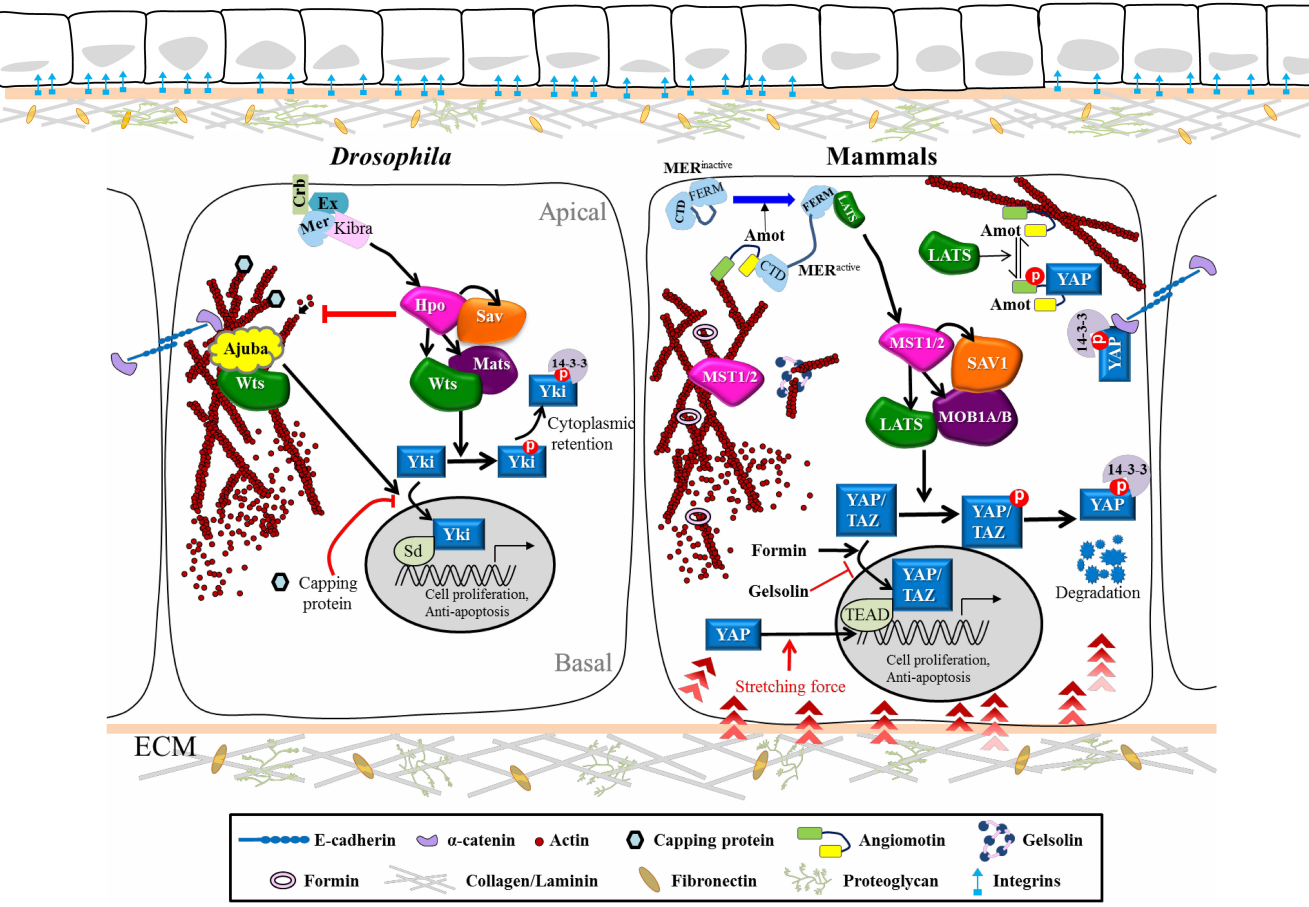
Cytoskeletal Regulation of the Hippo pathway in *Drosophila* and Mammals. Schematic diagram shows the core kinase cascade of the Hippo pathway and its interplay with upstream cytoskeletal regulators in *Drosophila* and mammals. Corresponding colors and shapes are used to indicate homologous components in both systems. When Hippo signaling is off, the nuclear effector Yki/YAP/TAZ can bind to the transcription factor Sd/TEAD to turn on target genes. Upon phosphorylation of Hpo/MST1/2 by upstream stimuli, Wts/LATS1/2 is activated and phosphorylates Yki/YAP/TAZ, which makes the later ones retain in the cytosol or further undergo degradation. The cytoskeletal regulators can also control the nuclear translocation of Yki/YAP/TAZ without their phosphorylation by Wts/LATS1/2. See text for further details. Hpo, Hippo; Sav, Salvador; Wts, Warts; Mats, Mob as a tumor suppressor; Yki, Yorkie; Ex, Expanded; Mer, Merlin; Crb, Crumbs; Sd, Scalloped.

Hippo (Hpo) signaling was initially identified through a genetic screen for cell size control in *Drosophila melanogaster*, revealing a conserved pathway that regulates organ size, cell fate, tissue homeostasis and tumor progression. The Hpo network is primarily a core kinase cascade, involving Hpo/mammalian Ste20-like kinases 1/2 (MST1/2), Salvador (Sav)/SAV1, Mats/MOB kinase activator 1A/B (MOB1A/B), and Warts (Wts)/large tumor suppressor kinase 1/2 (LATS1/2). This latter phosphorylates the transcription factor Yorki (Yki)/Yes-associated protein (YAP)/transcriptional coactivator with PDZ-binding motif (TAZ) to prevent its nuclear translocation, thereby blocking target gene activation (Ma et al., 2019) (Figure 1). Over the past decade, extensive research has established links between the Hpo pathway and organ size restriction, mainly based on the key roles of Hpo protein in controlling genes responsible for cell proliferation and anti-apoptosis. Therefore, how cells sense confluence to arrest the cell cycle and how mechanical force impacts on cell-cell contacts through junctional proteins is a critical facet of tissue architecture regulation during animal development. Disruption of epithelial homeostasis induced by overexpression of Yki or mutation of its upstream kinases allows tumor cells to freely proliferate and metastasize. Hpo signaling is unlike conventional ligand-receptor transduction pathways in that it integrates diverse upstream stimuli such as soluble factors, cell-cell adhesion, mechanical tension driven by the actin cytoskeleton, and force conveyed from the extra-cellular matrix (ECM). In this review, we summarize how the Hpo network functions and is regulated, the influence of its upstream inputs on physiology, and the contribution of its dysregulation to cancer metastasis. Since Hpo is an evolutionarily conserved pathway, components we describe herein include both *Drosophila* molecules and the corresponding vertebrate homologs.

## Components and Regulation of Hippo Signaling

The first mutant component of the Hpo pathway, *wts*, was retrieved from a forward genetic screen seeking tumor suppressors, with *hpo, sav* and *mats* being later identified based on the same phenotype of tissue overgrowth in mosaic mutant clones (Justice et al., 1995; Xu et al., 1995; Kango-Singh et al., 2002; Tapon et al., 2002; Harvey et al., 2003; Jia et al., 2003; Pantalacci et al., 2003; Lai et al., 2005; Zheng and Pan, 2019). Two of these components, *hpo* and *wts*, encode a serine/threonine kinase that belongs to the Ste-20 kinases and Nuclear Dbf2-related (NDR) family of proteins, respectively (Snigdha et al., 2019). Further studies have shown that the Hpo and Wts proteins both bind to the WW domain adaptor protein, Sav. Hpo phosphorylates Sav, which in turn phosphorylates Wts to activate the downstream Mats-Wts complex. The activated Wts kinase promotes Yki phosphorylation at Ser168, which induces association of Yki with 14-3-3 proteins, thereby reducing Yki nuclear localization (Oh and Irvine, 2008; Ren et al., 2010) (Figure 1). Very similar to the mechanism of Yki regulation in *Drosophila*, mammalian YAP is inactivated by LATS1/2 phosphorylation, resulting in 14-3-3 binding, retention in the cytosol, and subsequent protein degradation (Ma et al., 2019). Upon reduced Hpo signaling activity, the transcriptional coactivator Yki/YAP enters the nucleus where it works with DNA-binding transcription factors to activate genes involved in the cell cycle, cell proliferation and suppression of apoptosis (McClatchey and Yap, 2012). Recent live multiphoton microscopy analysis has further revealed that knock-in tagged Yki-Venus shuttles rapidly between the cytosol and the nucleus during interphase and then is enriched on mitotic chromatin, suggesting a role for Yki in regulating mitosis (Manning et al., 2018).

## Cell-Cell Contact and the Hippo Pathway

There is clear evidence illustrating that the cell cycle is arrested in G1 when cell growth reaches full confluence, which is defined as contact inhibition (Eagle and Levine, 1967). Hpo signaling has been implicated in contact inhibition because of YAP exclusion from the nucleus when cultured cells contact each other through adhesion molecules (Zhao et al., 2007). Expression of E-Cadherin in the breast cancer cell line MDA-MB-231 prevents nuclear localization of YAP. Antibody treatment against the extra-cellular domain of E-Cadherin inhibits proliferation of another breast cancer cell line (MCF-7) by redistributing YAP from the nucleus to the cytoplasm (Kim et al., 2011). α-Catenin that is attached to the cytoplasmic domain of E-Cadherin is the density sensor of skin cells, and it has been demonstrated that YAP localization and phosphorylation in keratinocytes is regulated by its interaction with α-Catenin. α-Catenin binds to phosphorylated YAP1 in the presence of 14-3-3, which prevents YAP1 activation and nuclear translocation. Overexpression of α-Catenin in low-density culture brings YAP to the plasma membrane, an outcome not observed upon overexpression of other components of adherens junctions such as E-Cadherin (Schlegelmilch et al., 2011). These findings suggest that α-Catenin is the mediator that transduces mechanical cues from E-Cadherin in fully confluent cultures, keeping YAP/α-Catenin/14-3-3 close to the plasma membrane and thereby preventing YAP activity in the nucleus.

Ajuba is a LIM domain scaffold protein that is known to be recruited to adherens junctions through its association with α-Catenin (Marie et al., 2003). Increasing tension mediated by cytoskeleton in *Drosophila* wing discs recruits Warts to adherens junctions by Ajuba in a tension-dependent manner, which can suppress Warts activity and hence lead to activation of Yki downstream genes (Rauskolb et al., 2014; Alegot et al., 2019). This scenario supports the idea that mechanical force may stimulate cell proliferation in cell cultures (Boggiano and Fehon, 2012; McClatchey and Yap, 2012).

## Mechanical Force Regulates Hippo Signaling

During tissue morphogenesis or organ development, cells constantly respond to mechanical stress from neighboring cells and the ECM, or to shear force when they migrate. Tension from different tissue geometries and degrees of matrix stiffness is transmitted through membrane receptors, the actin cytoskeleton and the nuclear membrane to affect gene expression within nuclei, which not only shapes tissue morphology but also determines cell cycle entry and cell fate specification. Recent research has unraveled the localizations of Hpo pathway components at cellular junctions, helping to more clearly depict how cellular morphology, the outside environment and F-actin architecture act together to control Hpo signaling activity (Figure 1). One cell culture study showed that mammalian MST1/2 is colocalized with filamentous actin and that disruption of actin stress fibers leads to MST1/2 activation (Densham et al., 2009). Studies in *Drosophila* have revealed that mutation in Capping proteins, a negative regulator of actin polymerization, causes F-actin accumulation, leading to upregulation of Yki target genes and tissue outgrowth in imaginal discs (Fernandez et al., 2011; Sansores-Garcia et al., 2011). Moreover, stress fibers or cell morphology itself can also promote YAP activity in mammalian cells in a LATS-dependent manner (Wada et al., 2011). Diaphanous, the mammalian Formin protein, facilitates actin filament assembly and also promotes YAP nuclear translocation, whereas the actin-severing components Gelsolin and Cofilin act as essential gate-keepers to antagonize the function of Yki/YAP in cell growth (Aragona et al., 2013; Gaspar and Tapon, 2014). According to these studies, actin polymerization positively regulates Yki/YAP activity. The upstream regulators relaying signals from membrane receptors to the cytoskeleton network were first identified in *Drosophila*, including Merlin (Mer) and Expanded (Ex) that coordinate cell proliferation. *ex* and *mer* double mutant cells exhibit tissue outgrowth and excessive BrdU staining, a phenotype similar to that caused by suppression of Hippo core kinase activity. Moreover, co-expression of Ex and Mer results in increased Warts phosphorylation, so Mer and Ex head the Hpo pathway (Hamaratoglu et al., 2006). Subsequently, *hpo, sav, mats, wts* or *ex* mutant clones were shown to display an F-actin accumulation phenotype, indicating that the Hpo pathway negatively controls actin filament assembly (Fernandez et al., 2011). However, overexpression of Moesin—an ERM (ezrin, radixin, moesin) protein that is localized at the apical domain of epithelia and promotes actin assembly—does not induce tissue outgrowth (Speck et al., 2003; Boggiano and Fehon, 2012). These observations suggest complex regulation of F-actin- and Hippo-dependent cell size control. Several lines of evidence indicate that the impact of cell morphology and mechanical stress on the Hpo pathway can also be eliminated by simply blocking Rho activity, which is required to build up the contractile network as cells react to a stiff matrix (Zhao et al., 2012; Aragona et al., 2013; Sun and Irvine, 2016). Interestingly, this regulation has been reported as being either LATS kinase-independent (Dupont et al., 2011) or -dependent (Wada et al., 2011; Zhao et al., 2012). Identification of Angiomotin (Amot) has clarified the relationship between F-actin and the Hpo pathway. Amot contains a conserved binding domain that can interact with F-actin or YAP. LATS-mediated phosphorylation of Amot prevents it from binding F-actin but promotes its association with YAP, leading to YAP retention in the cytosol (Mana-Capelli et al., 2014). This scenario potentially explains how the actin cytoskeleton is involved in LATS-independent regulation of YAP. In spatial control around the plasma membrane, Amot and the actin complex are regulated by Mer, a member of the ERM family of proteins that links the plasma membrane and actin filaments. ERM protein activity, including that of Mer, is negatively regulated by auto-inhibition via intramolecular binding between the N and C termini. Structure and function analyses have shown that interaction of Amot with Mer relieves such auto-inhibition and promotes Amot interaction with LATS through its C-terminal FERM domain (Li et al., 2015). Direct recruitment by Mer of LATS to the membrane further stimulates MST1/2-mediated phosphorylation of the LATS complex. Robust Mer-Wts/LATS binding by treatment with latrunculin B, an actin polymerization inhibitor, has been observed (Yin et al., 2013; Sun and Irvine, 2016), indicating that F-actin can potentiate this interaction. In addition, transmission of mechanical signals from the cytoskeleton to the nuclear membrane may also regulate Yki/YAP activity. Actomyosin filaments form a physical link with the nuclear envelope through the LINC (linker of nucleoskeleton and cytoskeleton) complex consisting of Lamin, Sun and Nesprin. Nesprin-1 and−2 are expressed ubiquitously in human. Both proteins encode an actin binding domain (ABD) at the N-terminus, a rod domain with several spectrin repeats that provide an interaction hub for Emerin and Lamin, and a C-terminal KASH domain inserted into the outer nuclear membrane (Janin and Gache, 2018). Nesprin-1 has recently been reported as playing a role in promoting nuclear localization of YAP in response to mechanical stretching to induce nuclear deformation in cultured cells (Driscoll et al., 2015). Furthermore, ECM-nuclear coupled stretching that flattens nuclei is sufficient to trigger nuclear import of YAP by diminishing the gating function of Nuclear Pore Complex (NPC) for selective transport (Elosegui-Artola et al., 2017). This latter study opens up a new avenue for investigating how mechanotransduction manipulates YAP nuclear transport, which may represent the mechanism by which Yki/YAP responds to mechanical cues when cells migrate through a crowded space in a short period of time.

## The Hippo Pathway in Cell Migration and Cancer Metastasis

For tumor cells to successfully metastasize, the epithelial to mesenchymal transition (EMT), invasion, intravasation and immune system-mediated evasion/extravasation must take place (Warren et al., 2018; Zanconato et al., 2019). Apart from its canonical role in tumor size control, Hpo signaling has been reported to contribute to cancer dissemination. Junction dissociation, apical-basal polarity loss and cytoskeleton network rearrangements are hallmarks of EMT. Several studies have demonstrated that, together with EMT-associated gene mutation, activation of YAP/TAZ or dysregulation of MST1/2 or LATS1/2 leads to cancer metastasis. Consistent with this scenario, knockdown of YAP/TAZ reverses the mesenchymal morphology of cancer cells (Lamar et al., 2012; Janse van Rensburg and Yang, 2016). In a screen for functional substitutes of KRAS, YAP was shown to promote survival of RAS-dependent colon cancer cell lines. Interestingly, YAP transcriptional profiling revealed that activation of the EMT-inducing transcription factor Fos promotes expression of mesenchymal genes and downregulates junctional proteins like E-Cadherin and Occludin (Shao et al., 2014). Moreover, expression of canonical EMT-inducing transcription factors such as Snail1/2, Slug, ZEB1 (Zinc Finger E-box-binding Homeobox 1) and Twist is driven by YAP in a variety of cancers (Warren et al., 2018). Taken together, these studies indicate that the Hpo pathway is involved in activation of the EMT program.

**Figure 2:**
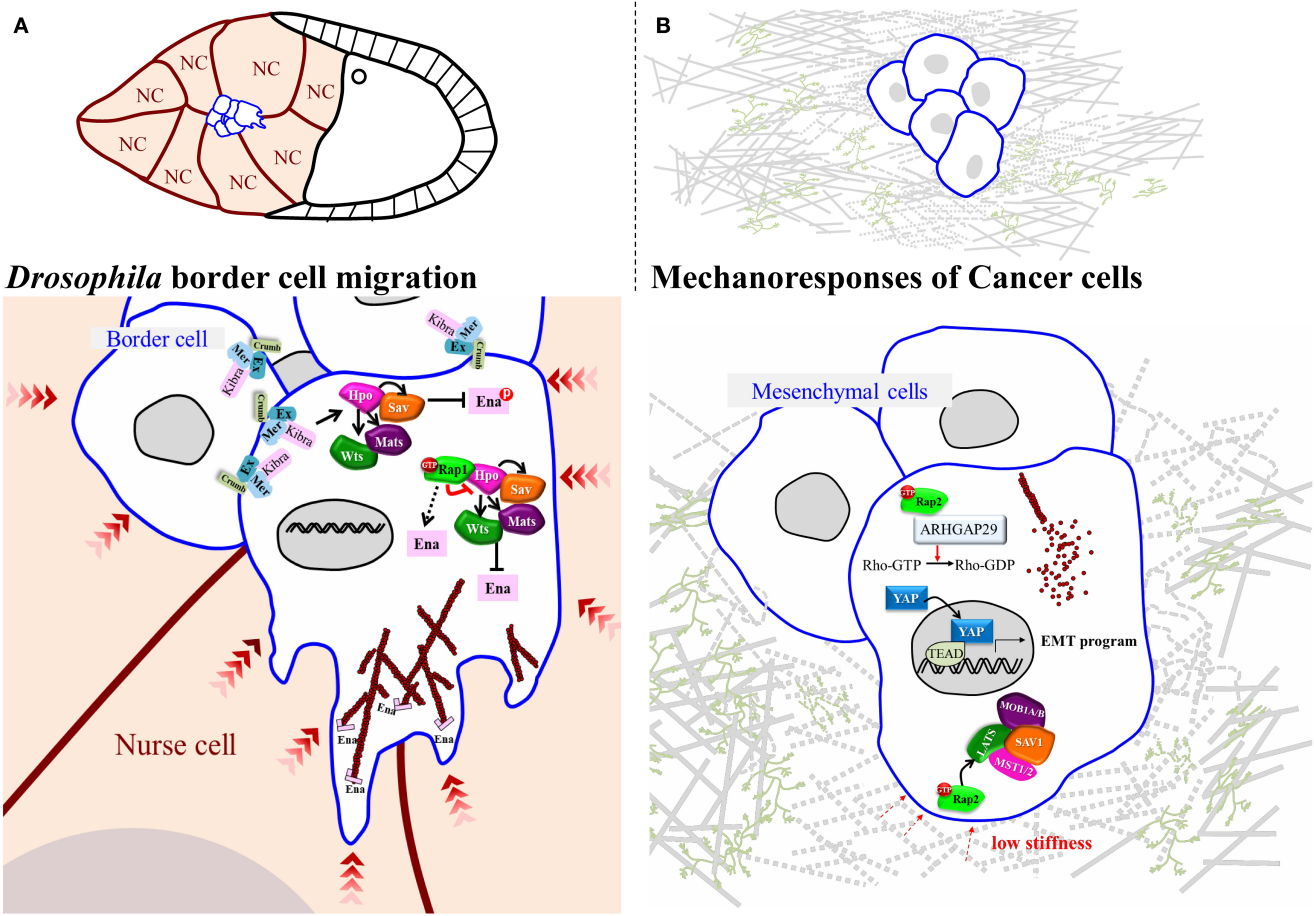
Regulatory Mechanisms for Small GTPase-Hippo-mediated Mechanoresponses in *Drosophila* and Mammals. **(A)** In *Drosophila* oogenesis, a small group of cells, named border cells, delaminate from the follicular epithelium and migrate through the germline cluster, nurse cells. During migration, the Hippo signaling is activated inside the migrating cluster where border cells form contact with each other. On the contrast, in the leading edge Rap1 mechanically suppresses Hippo pathway to promote actin-dependent protrusion. **(B)** In mammalian cells, at low stiffness Rap2 binds to and activates ARHGAP29 to turn on Hippo signaling, leading to actin depolymerization.

Apart from being activated by F-actin-mediated mechanical tension, YAP and Yki also regulate the actin dynamics that allow cancer cells to change shape and invade surrounding tissues (Lamar et al., 2012; Warren et al., 2018). In a model of circulating cancer cells, YAP has been shown to transcriptionally activate ARHGAP29, an inhibitor of RhoA, thereby reducing the rigidity of the cytoskeleton network by escalating G- and F-actin turnover (Qiao et al., 2017). This model has been well acknowledged for investigating the mechanism of distal metastasis in cancers (Nagrath et al., 2007; Massague and Obenauf, 2016), and it provides a clear insight into how YAP promotes cancer cell motility by affecting actin dynamics. Another study using a soft-polymer microfluidics system to mimic frictional force in blood vessels shows that YAP is activated by fluid shear stress and that it is required for fluid flow-induced cell motility (Lee et al., 2017). As a mechanosensor, YAP receives signals from surrounding tissues and counteracts this stress by activating ARHGAP29 to control actin dynamics, which is critical for cells to retain flexibility or to extend protrusions for metastasis. During *Drosophila* border cell migration, Hpo core kinases have been shown to polarize the distribution of F-actin, allowing cells to extend forward protrusions. At cell-cell contacts that do not extend cellular protrusions, Wts is required to phosphorylate Ena (Enabled)/VASP (vasodilator-stimulated phosphoprotein), inhibiting the function of the latter in promoting actin filament assembly (Lucas et al., 2013) (Figure 2A). This genetic analysis suggests that inactivation of Hpo signaling at the outer rim of the migrating cell cluster promotes protrusion formation. However, how Hpo signaling is downregulated remains unclear. An independent study that screened for Rap1 (Ras-related protein 1)-interacting proteins in this collective cell migration system identified Hpo as binding to active-form Rap1 (Chang et al., 2018). That interaction greatly impedes Hpo auto-phosphorylation, a critical step required for activation of Hpo signaling, which induces persistent protrusions at the leading edge of migrating cohorts (Figure 2A). Rap1, a Ras family small GTPase, has been demonstrated to regulate adherens junction formation and integrin signaling and to promote tumorigenesis in various cancers under Ras hyperactivity. Interestingly, Rap2, a close homolog of Rap1, is antagonized by Rap1 in endothelial cells (Pannekoek et al., 2013), and it serves as a mechanosensor to relay stress from ECM, as well as activating Lats1/2 in response to low stiffness (Meng et al., 2018) (Figure 2B). However, there is only one Rap1 gene in the *Drosophila melanogaster* genome, and studies of border cell migration in that system have exposed its role in suppressing Hpo signaling activity (Chang et al., 2018). Thus, Rap1 function might be context-dependent, so it is of great interest to establish if the underlying molecular mechanism is conserved in other *Drosophila* organs/tissues.

## Concluding Remarks

From a simple screen for cell size control in the fly, the Hpo signaling pathway has now expanded into a complex and highly conserved kinase network, comprising dozens of components and integrating diverse upstream signals that are required for many developmental, homeostatic and pathological processes. Our review has focused on summarizing how upstream signals, such as cellular junctions, cytoskeleton proteins and mechanical forces, link Hpo core kinase activity and YAP/Yki nuclear transport. The most challenging task is to unravel how all contributory mechanical and chemical cues act together to regulate the Hpo pathway *in vivo*. Hpo pathway activity is switched on and off by dynamic phosphorylation and dephosphorylation, so phosphatases are likely to play a vital role. Another unresolved issue is how mechanical stress is detected *in vivo* to influence Hpo signaling. Although analysis of knock-in tagged Yki-Venus illustrates the effect of tissue folding on Yki nuclear-cytoplasmic transport during morphogenesis (Fletcher et al., 2018; Manning et al., 2018), it remains difficult to identify direct spatial control on the Hpo pathway via mechanical force. The newly invented optogenetic tool, GFP-LARIAT (GFP-light-activated reversible inhibition by assembled trap) inhibits the function of GFP-tagged proteins by sequestering target proteins into a blue light-mediated multimeric module (Lee et al., 2014). This approach has been applied to spatiotemporally inhibit cytoskeleton proteins, myosin, and E-cadherin, among others (Qin et al., 2017). It would be worth applying this approach to disrupt the actin cytoskeleton network in a small region of the plasma membrane to investigate the impact of mechanical force on Hpo signaling, as it would allow visualization of the Hpo reporter in real time.

## Author Contributions

Y-CC and AJ co-wrote the manuscript. J-WW and C-WW accomplished all art work, critical reading, and comment on this manuscript.

### Conflict of Interest

The authors declare that the research was conducted in the absence of any commercial or financial relationships that could be construed as a potential conflict of interest.
